# Age-related reduction in ciprofol requirement for loss of consciousness during anesthesia induction: a prospective cohort study

**DOI:** 10.3389/fmed.2026.1755445

**Published:** 2026-03-31

**Authors:** Yu-Gang Lu, Fa-Qiang Zhang, Yi-Yu He, Tong Ding, Fang Deng, Wen Liu, Jin-Chao Song

**Affiliations:** 1 Department of Anesthesiology, Shanghai Pulmonary Hospital, Tongji University School of Medicine, Shanghai, China; 2 Department of Anesthesiology and Perioperative Medicine, Shanghai Fourth People's Hospital, Tongji University School of Medicine, Shanghai, China; 3 Department of Anesthesiology, Shidong Hospital Affiliated to University of Shanghai for Science and Technology, Shanghai, China

**Keywords:** anesthetics, ciprofol, elderly, intravenous anesthesia, loss of consciousness

## Abstract

**Background:**

Ciprofol, a novel intravenous anesthetic for procedural sedation and general anesthesia, characterized by rapid onset of action, lower rates of injection pain and effective sedation at lower doses, is a promising alternative to propofol. Further clinical evidence is needed on how to adjust the dosage regimen of ciprofol during anesthesia induction in elderly patients. This study aims to provide evidence-based dosing reference and investigate the correlation between patient age and the ciprofol requirement for loss of consciousness (LOC) during anesthesia induction.

**Methods:**

A total of 102 patients scheduled for elective surgery under general anesthesia were enrolled and grouped to Group A (51 patients, 18–64 years) and Group B (51 patients, ≥65 years) according to their age. All subjects received ciprofol (at a rate of 5 mg/kg/min) alone for inducing LOC, which was defined as loss of eyelash reflex and Modified Observer's Alertness/Sedation (MOAA/S) score of 1 or less. The dose of ciprofol and the time to loss of eyelash reflex for each subject were recorded.

**Results:**

Compared with patients in group A, patients in group B had a mean reduction in ciprofol requirement of 0.07 mg/kg and a mean reduction in the time to loss of eyelash reflex of 55.20 s. Bivariate linear correlation analysis showed that ciprofol requirement for LOC was significantly and positively correlated with albumin (*r* = 0.359; 95% CI = 0.177–0.518; *p* < 0.001), and negatively correlated with age, such that significant decline in ciprofol requirement with increasing age (*r* = −0.516; 95% CI = −0.645–−0.357; *p* < 0.001). Multivariable linear regression models were employed to assess the independent association between age and ciprofol requirement for LOC. After controlling for potential confounding variables, age remained a significant independent predictor of ciprofol requirement.

**Conclusion:**

The ciprofol requirement for LOC was significantly reduced in elderly patients. Our findings demonstrated that age serves as an independent factor influencing the ciprofol requirement for LOC during general anesthesia induction. These results suggest that the induction dose of ciprofol should be further reduced in elderly surgical patients, particularly those aged 75 years or older.

**Clinical trial registration:**

https://www.chictr.org.cn/; Identifier: ChiCTR2400093112.

## Introduction

Global population aging, coupled with advances in surgical techniques, has led to a substantial increase in elderly patients undergoing operative procedures. Studies indicate that surgical rates among individuals aged 65 years and older substantially exceed those of younger patients by approximately 300% (1, 2). Given current demographic trends, this disparity is anticipated to intensify in the coming decades. Consequently, anesthesiologists must frequently modify anesthetic protocols—including drug selection and dosage titration—to accommodate the physiological alterations associated with advanced age (3, 4). Nevertheless, robust evidence specifically guiding optimal anesthetic induction dosing for geriatric patients remains limited (5).

Ciprofol (HSK3486) is a new type of short-acting intravenous sedative. Structurally related to propofol, it produces sedative and anesthetic effects through potentiation of gamma-aminobutyric acid type A (GABAA) receptor activity. Compared with propofol, ciprofol offers several clinical advantages: more rapid onset of action, effective sedation at reduced doses, and decreased incidence of injection pain (2, 6, 7). Furthermore, accumulating evidence supports its efficacy and safety in elderly populations for anesthesia induction (8, 9). In a phase IIb trial, ciprofol 0.4–0.5 mg/kg produced equivalent sedation to propofol 2.0 mg/kg during colonoscopy in non-elderly adults, with a comparable safety profile (10).

Geriatric patients exhibit heightened sensitivity to sedative agents and often present with compromised cardiorespiratory function. Consequently, anesthesiologists typically reduce ciprofol induction doses from the standard 0.4–0.5 mg/kg to 0.2–0.4 mg/kg for patients aged over 65 years (8, 9, 11). However, the applicability of this dosing strategy to patients aged 75 years or older remains uncertain. Specifically, the optimal induction dose for this more vulnerable subgroup has not been clearly defined. To address this evidence gap, we designed the present study to investigate the appropriate ciprofol dose for elderly surgical patients and to characterize the influence of age on ciprofol requirements for achieving loss of consciousness (LOC).

## Patients and methods

### Participants

We conducted a prospective cohort study following the Declaration of Helsinki from December 2024 to February 2025. The study was approved by the Institutional Research Ethics Committee of Shidong Hospital. Patients aged 18 years and over, with American Society of Anesthesiologists (ASA) physical status I to II and body mass index (BMI) between 18 and 29 kg/m^2^, scheduled for general surgery or orthopedic surgery under general anesthesia were considered eligible. Written informed consent was obtained from all patients.

Exclusion criteria were the following: allergic to soy products or propofol; taking hypnotics, opioid analgesic or antianxiety agents; significant cardiovascular disease, defined as ejection fraction (EF) < 55%, New York Heart Association (NYHA) functional class II-IV, or uncontrolled arrhythmia, severe respiratory disease, renal or metabolic diseases; history of neurological or psychiatric disorders; could not complete the informed consent procedure independently.

### Study protocol

A total of 102 patients who met the inclusion and the exclusion criteria were divided into two groups, Group A (51 patients, 18–64 years), Group B (51 patients, ≥65 years), according to age. After being transferred to the operating room, a peripheral intravenous catheter was placed, and Ringer's solution infusion was started at 16–18 ml/kg/h in the operating room. Non-invasive blood pressure, heart rate (HR), electrocardiogram (ECG), pulse oxygen saturation (SpO_2_) and end-tidal carbon dioxide (EtCO_2_) were monitored continuously throughout the operation. After 5 min of preoxygenation (oxygen flow 5 L/min), ciprofol (Shenyang Haisco Pharmaceutical Co., Ltd., Shenyang, China) was delivered by a Graseby 3500 syringe pump (Smiths Medical, UK) at a rate of 5 mg/kg/min until the LOC occurred. LOC was defined as loss of eyelash reflex and Modified Observer's Alertness/Sedation (MOAA/S) score of 1 or less. The assessment of the loss of eyelash reflex and verbal response was carried out every 10 s after 1.5 min of continuous infusion of ciprofol. An anesthesiologist assistant, who was blinded to this study, performed the above reflex assessment and finally determined the end point of titration. Meanwhile, the dose of ciprofol (Ciprofol requirement) and the time to loss of eyelash reflex for each patient were recorded. Upon successful induction, 0.4–0.6 ug/kg of sufentanil (Yichang Humanwell Pharmaceutical Co., Ltd., Yichang, China) and 0.2 mg/kg of cisatracurium (Nanjing Jianyou Biochemical Pharmaceutical Co., Ltd., Nanjing, China) were administered, and endotracheal intubation was performed 3 min later. General anesthesia was maintained using remifentanil (0.2–0.3 μg/kg/min) and sevoflurane (1.2%−2.5%).

Perioperative variables included in the analysis were sex, BMI, ejection fraction (EF), total bilirubin (TBIL), albumin (ALB), alanine aminotransferase (ALT), aspartate aminotransferase (AST), serum creatinine (Scr), blood urea nitrogen (BUN), international normalized ratio (INR) and type of surgery. Blood samples were drawn between 07:00 and 08:00 on the morning, 1–2 days prior to surgery. Hemodynamic parameters including systolic blood pressure (SBP), mean arterial pressure (MAP) and HR at five different time points (T0, before ciprofol administration; T1, LOC; T2, 3 min after the administration of sufentanil and cisatracurium; T3, 1 min after intubation; T4, 5 min after intubation; T5, 10 min after intubation) were recorded. Postinduction hypotension (PIH) was defined as a MAP < 65 mmHg or a MAP reduction of more than 20% from baseline. Any MAP dropped by a MAP < 65 mmHg or by more than 20% from baseline for at least 30 s within the first 10 min after induction triggered the administration of intravenous ephedrine boluses (5 mg). Bradycardia was defined as a HR < 50 bpm persisting more than 30 s and was treated with intravenous atropine boluses (0.5 mg). Ventilatory frequency was adjusted to maintain EtCO2 concentration at 35–45 mmHg.

### Statistical analysis

Group sample size calculation was based on a difference of ciprofol requirement for LOC observed in a pilot experiment, in which the mean ciprofol requirement was 0.36 ± 0.11 mg/kg (*n* = 10) in the adult group and 0.31 ± 0.06 mg/kg (*n* = 10) in the elderly group. Using a 1:1 allocation ratio, the determined sample size of 51 patients per group fulfilled the requirement with α = 0.05, power = 0.80, standard deviation = 0.09.

Continuous variables were expressed as mean ± standard deviation (SD) (age, height, weight, BMI, or albumin level), or median (interquartile range, IQR) (EF, INR, total bilirubin, ALT, AST, Scr, or BUN levels) with normal or non-normal distributions, and categorical data were summarized as numbers (percentages) (gender and surgical procedures). Statistical differences between groups were analyzed using the student's *t*-test (age, height, weight, BMI, or albumin level) or Mann–Whitney *U* test (EF, INR, total bilirubin, ALT, AST, Scr, or BUN levels), respectively. The chi-square test was applied for categorical variables analysis (gender and surgical procedures). The correlations between the ciprofol dose requirement and other parameters (age, BMI, EF, INR, albumin, total bilirubin, ALT, AST, Scr, or BUN levels) were evaluated using Pearson's or Spearman's correlation coefficients to account for linear and non-linear trends. The linearity between age and ciprofol requirement after adjusting for potential confounders was visually assessed using smooth curve fitting.

A two-sided *p* value < 0.05 was deemed statistically significant for all tests. Analyses were conducted with PASS (version 15.0), SPSS software (version 26.0) and R Statistical Language (version 4.0.5).

### Sensitivity analysis

To evaluate the potential confounders, we specified univariate or multivariate linear models to assess whether age was independently correlated with ciprofol requirement. Model 1 (unadjusted) was a univariate model. Model 2 was adjusted for gender and BMI. Model 3 was adjusted for gender, BMI, albumin, ALT, Scr, and BUN levels. Additionally, sensitivity analysis was applied to assess the relationship of age with ciprofol requirement according to gender, BMI, albumin, ALT, Scr, and BUN levels, as described in previous studies. We further evaluated the percent change from baseline in SBP, MBP or HR between groups at different time points.

## Results

**Figure 1:**
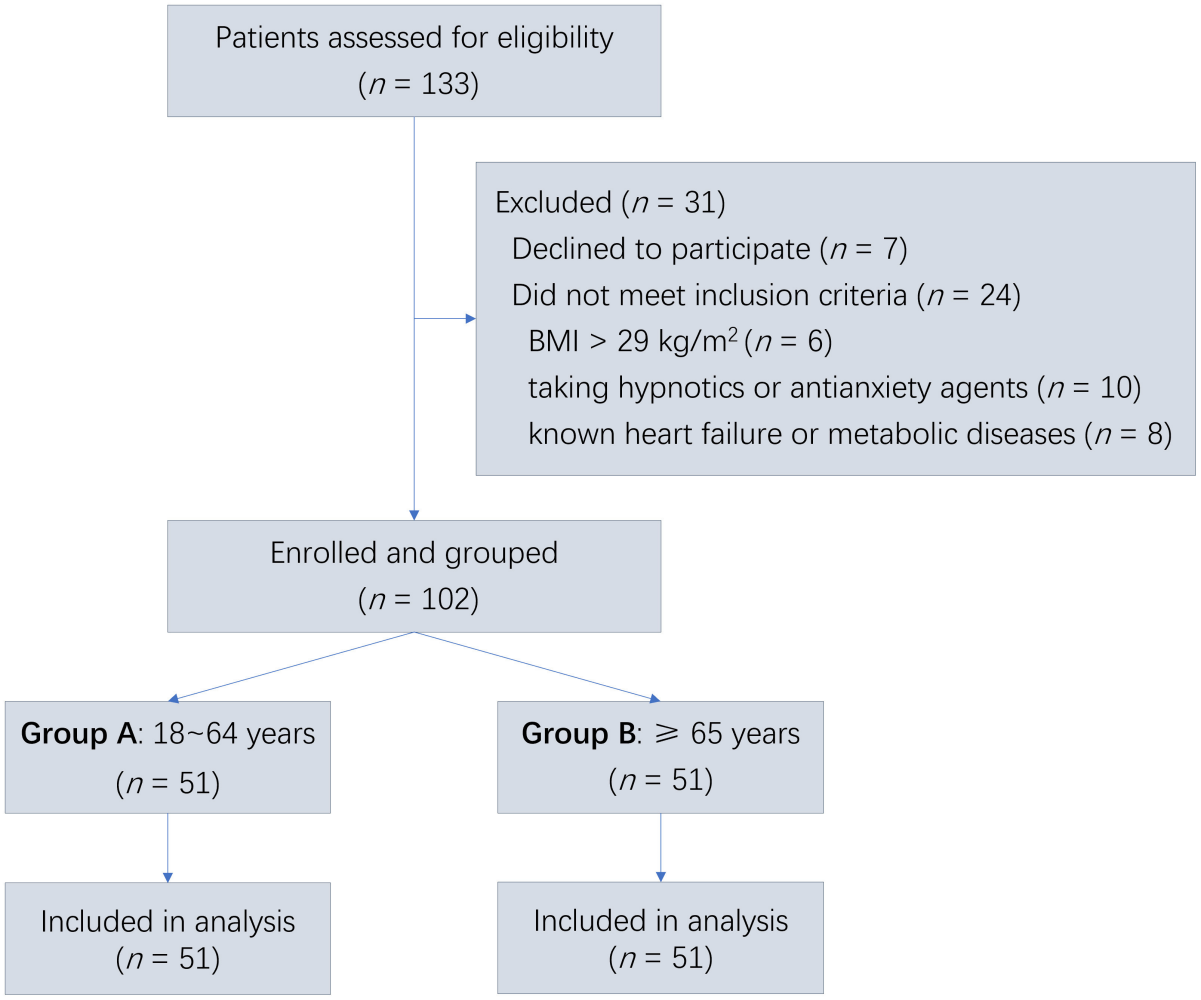
Study flowchart of patient enrollment and group allocation. Patients aged 18–64 years were assigned to Group A (younger group), while those aged ≥65 years were assigned to Group B (elderly group). All patients received ciprofol titrated to achieve loss of consciousness (LOC) during general anesthesia induction. BMI, body mass index.

A total of 133 patients were initially assessed for eligibility, 31 of whom were excluded (7 declined to participate, 24 did not meet inclusion criteria). Finally, 102 patients were enrolled and grouped to Group A (*n* = 51) and Group B (*n* = 51) according to their age. Following the intention-to-treat principle, data analysis was conducted up to the cut-off point, resulting in a full analysis set comprising 102 individuals (51 in each group) (Figure 1).

The baseline characteristics of patients are shown in Table 1. Of the 102 patients (mean age = 64.69 ± 12.81 years), 55 (53.92%) were female. No differences were observed in gender, BMI, TBIL, ALT, AST, Scr and BUN between the two groups. However, compared with patients in group A, patients in group B have lower EF values (*p* = 0.004) and albumin levels (*p* < 0.001), whereas INR levels (*p* = 0.035) were higher.

**Table 1:** Baseline demographic, anthropometric, and biochemical characteristics of surgical patients by age group.

Variables	Overall (*n* = 102)	Group A (*n* = 51)	Group B (*n* = 51)	*P* value
Age, mean (SD), y	64.69 (12.81)	54.39 (9.27)	74.98 (5.42)	< 0.001
Male, n (%)	47 (46.08)	20 (39.22)	27 (52.94)	0.164
Height, mean (SD), cm	164.00 (8.00)	165.00 (8.00)	164.00 (8.00)	0.360
Weight, mean (SD), kg	64.42 (10.28)	66.70 (11.45)	62.14 (8.47)	0.024
BMI, mean (SD), kg/m^2^	23.90 (3.69)	24.50 (3.99)	23.31 (3.29)	0.103
EF, median (IQR), %	62.00 (62.00, 63.00)	62.00 (62.00, 64.00)	62.00 (61.00, 62.50)	0.004
INR, median (IQR), unit	0.98 (0.92, 1.02)	0.96 (0.89, 1.00)	0.99 (0.95, 1.03)	0.035
Liver function				
Albumin, mean (SD), g/L	41.48 (5.18)	43.71 (4.82)	39.26 (4.57)	< 0.001
Total bilirubin, median (IQR), μmol/L	12.85 (9.30, 16.35)	13.80 (8.90, 16.70)	12.30 (9.40, 15.95)	0.527
ALT, median (IQR), U/L	19.00 (16.00, 24.00)	19.00 (17.00, 24.50)	19.00 (13.50, 24.00)	0.455
AST, median (IQR), U/L	23.50 (19.25, 28.75)	23.00 (19.00, 27.00)	24.00 (20.50, 29.00)	0.412
Kidney function				
Scr, median (IQR), μmol/L	63.85 (55.02, 76.42)	60.80 (53.25, 73.85)	68.50 (58.65, 81.95)	0.050
BUN, median (IQR), mmol/L	5.38 (4.43, 6.40)	5.12 (4.22, 6.01)	5.47 (5.00, 6.78)	0.067
Procedures, *n* (%)				
Gynecological surgery	15 (14.71)	12 (23.53)	3 (5.88)	0.033
Orthopedic surgery	25 (24.51)	11 (21.57)	14 (27.45)	
Urology surgery	12 (11.76)	3 (5.88)	9 (17.65)	
General surgery	50 (49.02)	25 (49.02)	25 (49.02)	

Group A: patients aged 18–64 years; Group B: patients aged ≥ 65 years. Data are presented as mean (standard deviation), median (interquartile range), or *n* (%). Statistical comparisons performed using independent *t*-test, Mann–Whitney *U* test, or chi-square test as appropriate. BMI, body mass index; EF, ejection fraction; INR, international normalized ratio; ALT, alanine aminotransferase; AST, aspartate aminotransferase; Scr, serum creatinine; BUN, blood urea nitrogen.

**Figure 2:**
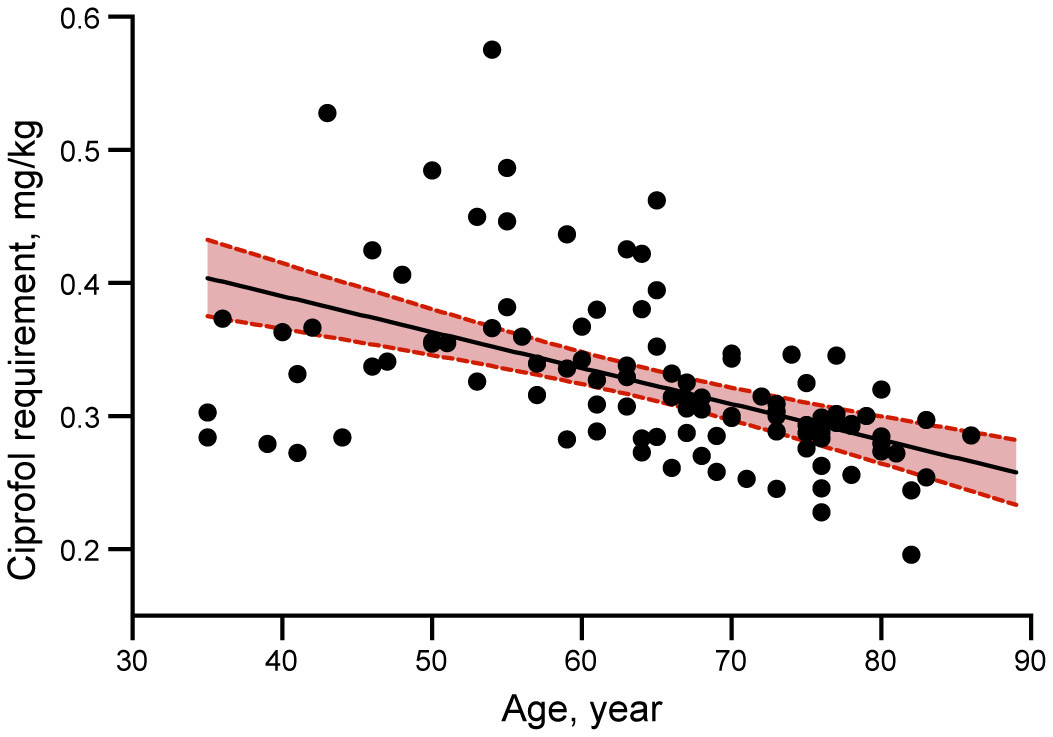
Adjusted dose-response linear relationship between patient age and ciprofol dosage required for loss of consciousness (LOC) during general anesthesia induction. The analysis demonstrates a significant inverse correlation (β = −0.002 to −0.003 per year, *p* < 0.001), with older patients requiring lower ciprofol doses. Model adjusted for gender, body mass index (BMI), albumin, alanine aminotransferase (ALT), serum creatinine (Scr), and blood urea nitrogen (BUN). Shaded area represents 95% confidence interval.

Differences in sedative and anesthetic effects between groups are shown in Table 2. There were significant differences in ciprofol requirement (*p* < 0.001) and time to loss of eyelash reflex (*p* < 0.001) between the two groups. Compared with patients in group A, patients in group B had a mean reduction in ciprofol requirement of 0.07 mg/kg and a mean reduction in time to loss of eyelash reflex of 55.20 s. Additionally, to further verify whether the relation between age and ciprofol requirement was linear, the estimated dose-response curve was fitted. There was a continuous linear decreasing trend and statistical significance between ciprofol requirement and age after adjusting for gender, BMI, albumin, ALT, Scr and BUN (Figure 2).

**Table 2:** Ciprofol dosage requirements and induction characteristics in younger vs. elderly surgical patients.

Variable	Overall (*n* = 102)	Group A (*n* = 51)	Group B (*n* = 51)	*P* value
Ciprofol dose, mean (SD), mg/kg	0.32 (0.07)	0.36 (0.07)	0.29 (0.04)	< 0.001
Time to loss of eyelash reflex, mean (SD), s	232.80 (48.60)	260.40 (49.20)	205.20 (27.60)	< 0.001

Group A (aged 18–64 years) received weight-based ciprofol titration to achieve loss of consciousness (LOC), with a mean dose of 0.36 (0.07) mg/kg. Group B (aged ≥ 65 years) required significantly lower doses [0.29 (0.04) mg/kg, *p* < 0.001], representing a 19.4% reduction. Time to LOC (loss of eyelash reflex) was also significantly shorter in elderly patients. Data presented as mean (standard deviation).

Bivariate linear correlation analysis showed that ciprofol requirement was significantly and positively correlated with albumin (*r* = 0.359; 95% CI = 0.177–0.518; *p* < 0.001), and negatively correlated with age, such that significant decline in ciprofol requirement with increasing age (*r* = −0.516; 95% CI = −0.645 to −0.357; *p* < 0.001). Other coefficients were not significantly correlated with ciprofol requirements (Table 3).

**Table 3:** Univariate correlations between ciprofol dosage requirement for loss of consciousness and patient characteristics.

Variable	Ciprofol requirement, mg/kg		
	*R*	95% CI	*p* value
Age, y	−0.516	−0.645 to −0.357	< 0.001
BMI, kg/m^2^	−0.026	−0.220 to 0.169	0.793
EF, %	0.092	−0.104 to 0.282	0.355
INR, unit	−0.005	−0.200 to 0.189	0.958
Albumin, g/L	0.359	0.177 to 0.518	< 0.001
Total bilirubin, μmol/L	0.186	−0.009 to 0.367	0.062
ALT, U/L	0.054	−0.142 to 0.246	0.589
AST, U/L	−0.095	−0.284 to 0.101	0.341
Scr, μmol/L	0.106	−0.090 to 0.295	0.287
BUN, mmol/L	0.179	−0.016 to 0.361	0.071

Pearson's correlation coefficient (*R*) was used for normally distributed continuous variables; Spearman's rho for non-normally distributed variables. Age showed the strongest inverse correlation with ciprofol requirement (*R* = 0.516, 95% CI: 0.645 to 0.357, *P* < 0.001), while albumin showed a positive correlation (*R* = 0.359, *P* < 0.001). BMI, body mass index; EF, ejection fraction; INR, international normalized ratio; ALT, alanine aminotransferase; AST, aspartate aminotransferase; Scr, serum creatinine; BUN, blood urea nitrogen; CI, confidence interval.

Multivariable linear regression analysis was adopted to determine the independent impact of age on ciprofol dosage requirements. When ciprofol dosage was incorporated into the models as a continuous variable, advanced age demonstrated a significant association with reduced ciprofol requirements (Table 4). Model 1, as an unadjusted baseline model incorporating only the age variable, was designed to preliminarily evaluate the influence of age on ciprofol dosage. Following the results of univariate linear regression analyses, both gender and BMI were identified as statistically significant predictors (*p* < 0.05) and were therefore incorporated into Model 2. Based on prior research findings (12–14), Model 3 incorporates indicators such as ALB, ALT, Scr, and BUN. These variables were selected due to their established strong correlation with hepatic and renal function, as well as their demonstrated influence on ciprofol pharmacokinetics. Our results revealed that per 1 SD increase in age was associated with a decrease in ciprofol requirement of 0.003 mg/kg (95% CI = −0.990 to −0.002; *p* < 0.001) in Model 1. After adjusting for potential confounding variables, we found that each 1 SD increase in age was associated with a reduction in ciprofol dosage of 0.003 mg/kg (95% CI = −0.990 to −0.002; *p* < 0.001) in Model 2, and a comparable decrease of 0.002 mg/kg (95% CI = −0.990 to −0.001; *p* < 0.001) in Model 3 (Table 4). Compared with patients in Group A, elderly patients in Group B exhibited a significantly reduced ciprofol requirement for LOC in the unadjusted model (β= −0.077; 95% CI = −0.098 to −0.055; *p* < 0.001). This association remained statistically significant after adjusting for gender and BMI in Model 2 (β = −0.077; 95% CI = −0.098 to −0.055; *p* < 0.001), and further persisted following additional adjustment for ALB, ALT, Scr, and BUN levels in Model 3 (β = −0.069; 95% CI = −0.092 to −0.045; *p* < 0.001).

**Table 4:** Multivariate linear regression analysis of age as an independent predictor of ciprofol dosage requirement.

Age, year	Model 1		Model 2		Model 3	
	β (95% CI)	*p* value	β (95% CI)	*p* value	β (95% CI)	*p* value
Per 1 year	−0.003 (−0.990 to −0.002)	< 0.001	−0.003 (−0.990 to −0.002)	< 0.001	−0.002 (−0.990 to −0.001)	< 0.001
Group A	0.000 (reference)		0.000 (reference)		0.000 (reference)	
Group B	−0.077 (−0.098 to −0.055)	< 0.001	−0.077 (−0.098 to −0.055)	< 0.001	−0.069 (−0.092 to −0.045)	< 0.001

The dependent variable was ciprofol dose required to achieve loss of consciousness (mg/kg). Model 1: unadjusted; Model 2: adjusted for gender and BMI; Model 3: fully adjusted for gender, BMI, albumin, ALT, Scr, and BUN. Age remained a significant independent predictor across all models, with each 1-year increase associated with a 0.002–0.003 mg/kg reduction in ciprofol requirement. Group B (≥65 years) required 0.069–0.077 mg/kg less ciprofol than Group A (18–64 years) after multivariate adjustment. β, standardized regression coefficient; CI, confidence interval; BMI, body mass index; ALT, alanine aminotransferase; Scr, serum creatinine; BUN, blood urea nitrogen.

**Figure 3:**
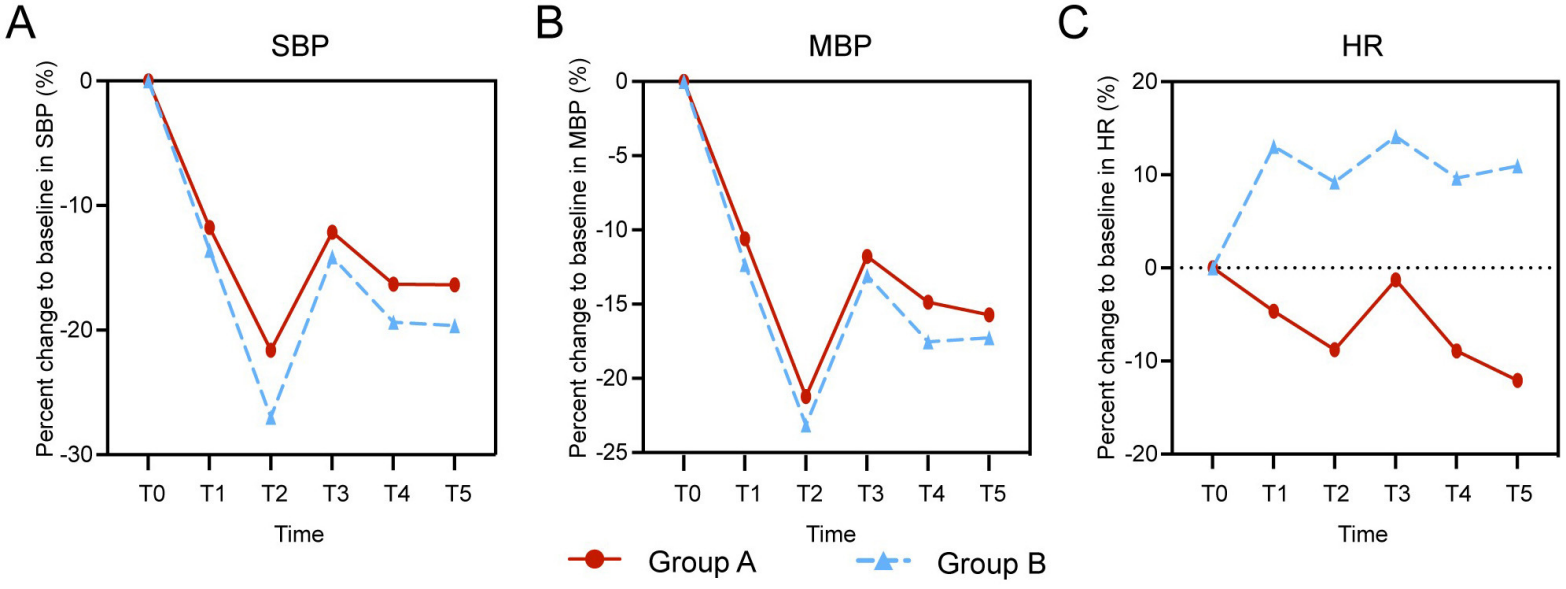
Hemodynamic changes during general anesthesia induction with age-stratified ciprofol dosing. Percentage changes from baseline in **(A)** systolic blood pressure (SBP), **(B)** mean arterial pressure (MAP), and **(C)** heart rate (HR) are shown for Group A (aged 18–64 years, *n* = 51, red circles) and Group B (aged ≥ 65 years, *n* = 51, blue triangle). Time points: T0, baseline; T1, loss of consciousness (LOC) after ciprofol administration; T2, 3 min after sufentanil and cisatracurium; T3, 1 min after tracheal intubation; T4, 5 min post-intubation; T5, 10 min post-induction. Data presented as mean. Elderly patients (Group B) received 19.4% lower ciprofol doses (0.29 vs. 0.36 mg/kg) yet exhibited comparable hemodynamic stability.

Following the induction of general anesthesia, the SBP and MAP of patients in both groups exhibited a progressive decline, with the most pronounced decrease observed at time point T2 (3 min following administration of sufentanil and cisatracurium). After intubation, both SBP and MAP exhibited varying degrees of rebound and stabilized within 5–10 min. Nevertheless, no statistically significant difference in the percentage change from baseline was observed between the two groups (SBP: T1, *p* = 0.865; T2, *p* = 0.179; T3, *p* = 0.991; T4, *p* = 0.842; T5, *p* = 0.807; MAP: T1, *p* = 0.959; T2, *p* = 0.983; T3, *p* = 0.999; T4, *p* = 0.930; T5, *p* = 0.995) (Figures 3A, B). Compared with the T0 baseline level, the HR of patients in Group A showed a progressive declining trend after anesthesia induction, whereas the HR of patients in Group B exhibited an increasing trend. Notably, within the 5–10 min time window following endotracheal intubation, the difference in HR changes between the two groups was statistically significant (HR: T1, *p* = 0.067; T2, *p* = 0.059; T3, *p* = 0.154; T4, *p* = 0.048; T5, *p* = 0.006) (Figure 3C).

## Discussion

In the present study, we evaluated the effective dose of ciprofol required to achieve LOC during the induction of general anesthesia in elderly surgical patients. Our findings demonstrated a significant inverse correlation between patient age and the ciprofol dosage required for LOC. Furthermore, multivariate analysis confirmed that age served as an independent and statistically significant predictor of ciprofol requirements for LOC. These results suggest that anesthesia induction protocols should incorporate age-specific dosing adjustments, with particular emphasis on further dose reduction for elderly patients, especially those aged 75 years and above.

Elderly patients often exhibit diminished cardiac reserve and multiple comorbidities. To optimize anesthesia safety and efficacy in this population, individualized protocols prioritizing hemodynamic stability and tailored induction strategies are essential (15). Research indicates that ciprofol was well tolerated at treatment doses of 0.2, 0.3, and 0.4 mg/kg in healthy Chinese elderly subjects and 0.4 mg/kg in non-elderly individuals (11). A randomized, controlled trial evaluating the efficacy and safety of varying ciprofol dosages for the induction of general anesthesia in elderly patients demonstrated that a ciprofol dose of 0.3 mg/kg exhibits both favorable safety and efficacy profiles for the indication (15). In our study cohort, the effective dose of ciprofol for LOC in patients (≥65 years) was 0.29 mg/kg, which is 19.4% lower than that for patients aged < 64 years (Table 2). Our findings are in line with previous studies (10, 11, 15). Furthermore, our results show that each 1 SD increase in age was associated with a reduction of 0.002–0.003 mg/kg in ciprofol dosage requirement for LOC (Table 4). Based on age-stratified analysis, our findings demonstrate a significant age-dependent reduction in the ciprofol dosage required to achieve LOC, with each successive decade of age showing a marked decrease in ciprofol requirements (Figure 1). These findings validate the current guidelines advocating for minimal intravenous sedative administration and further recommend reducing the induction dose of ciprofol specifically for patients aged 75 years and above.

In our study, the bivariate linear correlation analysis revealed a positive correlation between ciprofol requirement and serum albumin levels. This correlation is closely associated with the pharmacokinetic and pharmacodynamic characteristics of ciprofol: approximately 95% of ciprofol binds to plasma proteins, with serum albumin serving as its primary carrier (16). Studies show ciprofol is 4–5 times more potent than propofol (e.g., 0.4 mg/kg ciprofol ≈ 2.0 mg/kg propofol) (2). The cyclopropyl group in ciprofol endows it with higher lipid solubility than propofol, which not only enhances its ability to cross cell membranes but also strengthens its binding affinity to plasma proteins such as albumin. Notably, albumin binding directly regulates the pharmacodynamic effects of ciprofol by limiting its diffusion into target tissues (e.g., the brain). For patients with normal or high albumin levels, a higher dose of ciprofol is required to compensate for the reduced unbound drug fraction, thereby ensuring the desired anesthetic effect. In contrast, hypoalbuminemia (e.g., in patients with liver disease) leads to an increase in the free drug concentration of ciprofol, which means lower doses may be sufficient to achieve effective anesthesia (12). This is particularly critical because the increased free drug fraction in hypoalbuminemic patients may also exacerbate adverse effects such as hypotension and respiratory depression, highlighting the necessity of dose adjustment. Furthermore, albumin binding may affect the metabolism of ciprofol: it can slow down the metabolism of ciprofol (which is metabolized to propofol and formaldehyde), thereby prolonging the drug's action duration while requiring higher initial doses for rapid induction of anesthesia. A recent trial exploring ciprofol dosing for gastrointestinal endoscopy in obese patients (BMI ≥ 28 kg/m^2^) also emphasized that individualized dosing based on physiological variables, including albumin levels, is crucial to ensure the safety and efficacy of ciprofol (17). The marked difference in albumin values observed in our study is not only closely associated with the variability of ciprofol dosage requirements but also exerts a significant impact on its pharmacodynamic effects. Therefore, monitoring serum albumin levels is a key clinical measure to guide personalized ciprofol dosing, particularly in special populations with altered protein binding capacity, such as the elderly, critically ill patients, and those with liver dysfunction.

Our primary objective of this study was to examine the effect of age on the ciprofol dosage required to achieve LOC during anesthesia induction in elderly patients. To account for potential confounding variables, we incorporated factors such as BMI, EF, INR, serum albumin levels, total bilirubin levels, ALT, AST, Scr and BUN into our analytical models (Table 3). After adjustment for these factors, age remained an independent factor. In light of the greater sensitivity of the elderly to ciprofol, our findings are consistent with previous studies (11, 18, 19). In contrast to prior research paradigms, we focused on elderly surgical patients aged 65 years or older and sought to eliminate interference from concomitant medications, comorbidities, and renal insufficiency. Notably, this geriatric surgical population represents a distinct group, commonly presenting with cardiopulmonary dysfunction, metabolic diseases, and nervous system dysfunction, etc. (3). Advancing age reflects the physiological changes inherent in the senescence process, rather than constituting a pathological state *per se*. Consequently, this age-related progression progressively compromises the organism's capacity to maintain homeostatic balance, particularly under conditions of physiological stress (3, 20, 21). Meanwhile, existing research has consistently demonstrated that advanced age serves as an independent prognostic factor adversely affecting outcomes across a wide spectrum of surgical interventions. Therefore, anesthetists should maintain heightened awareness of the physiological changes associated with aging in order to optimize perioperative management strategies for elderly surgical patients. As a novel intravenous sedative, ciprofol demonstrates excellent clinical efficacy and a favorable safety profile. It offers superior circulatory stability and significantly fewer respiratory complications, making it a promising alternative to propofol for elderly patients undergoing elective surgery (2).

Cardiovascular responses serve as highly sensitive physiological indicators of noxious stimuli, particularly during procedures such as endotracheal intubation under general anesthesia (22, 23). Propofol induces depression of HR and blood pressure (BP) via GABAA receptor-mediated modulation of autonomic regulatory mechanisms within the brainstem (24, 25), highlighting the clinical significance of evaluating hemodynamic effects during and after the administration of ciprofol or propofol. A meta-analysis aimed at evaluating the efficacy and safety of ciprofol in the perioperative management of elderly patients demonstrated a significantly lower incidence of bradycardia in the ciprofol group (2). This suggests that ciprofol offers advantages for patients at high risk of perioperative circulatory complications, such as the elderly. It may be attributed to ciprofol's pharmacological properties, including its tighter binding to GABAA receptors, lower lipophilicity, and more uniform distribution in organs and tissues (26). In the present study, The SBP and MBP of the patients exhibited a gradual decline following ciprofol infusion, which further decreased upon administration of muscle relaxants and analgesics. Endotracheal intubation reversed the declining trend of BP, and stabilized within 10 min. Notably, elderly patients exhibited a certain degree of HR elevation following the induction with ciprofol, in contrast to the HR decrease observed in the non-elderly patients. Concerns on hemodynamic depression of cipofol in elderly patients prompted us to use lower doses of analgesics to implement balanced anesthesia. This may explain the trend of HR changes in elderly patients during anesthesia induction; however, additional investigative efforts are required to elucidate the underlying pathophysiological mechanisms. Therefore, during the induction of anesthesia in elderly patients, in addition to the dosage of ciprofol, we must also focus on the rational control of administration speed and the optimal selection of combination drug regimens.

This study had several limitations. First, this was a single-center study, which may be subject to potential selection biases related to patient race, surgical procedure type, cipofol infusion rate and general anesthesia induction protocol. Second, our study exclusively enrolled patients classified as ASA I–II. While this inclusion criterion effectively minimized potential confounding factors associated with comorbid conditions such as diabetes mellitus or hypertension. Our linear age-dose model should not be extrapolated to ASA III–IV cases without further validation. Third, differences in anesthesia induction protocols may lead to significant variations in hemodynamic fluctuations among surgical patients. Furthermore, this study did not evaluate the effects of ciprofol over the entire intraoperative period or its impact on long-term postoperative outcomes, including cognitive function and complication rates. A comprehensive assessment of these parameters is crucial for a thorough understanding of ciprofol's clinical efficacy and therapeutic value. Further research employing larger sample sizes, varied drug infusion rates, narrower age intervals (e.g., 10-year increments), and extended follow-up periods will be essential to more precisely delineate the correlation between age and ciprofol induction dosage in elderly surgical patients.

In conclusion, this study establishes a significant and independent correlation between age and the dosage of ciprofol required to induce loss of consciousness (LOC). The ciprofol dosage should be individualized in elderly patients, particularly those aged over 75 years, as this tailored approach may confer clinical benefits to this vulnerable population. Further large-scale clinical trials are needed to validate this conclusion and to verify whether this will improve the perioperative prognosis of geriatric patients.

To translate these findings into clinical practice, we recommend the following: (1) For patients aged 75 years and above, consider initiating induction with a ciprofol dose of 0.25–0.28 mg/kg (approximately 15%−20% reduction from standard dosing), followed by careful titration based on patient response and hemodynamic monitoring; (2) Implement age-stratified dosing protocols within institutional anesthesia guidelines, incorporating mandatory hemodynamic monitoring during the induction phase given the diminished cardiac reserve in this population; and (3) For very elderly patients (≥80 years) or those with significant comorbidities, adopt a “start low, go slow” strategy with even further dose reductions (e.g., 0.20–0.25 mg/kg) and ensure immediate availability of vasopressor support.

## Data Availability

The raw data supporting the conclusions of this article will be made available by the authors, without undue reservation.

## References

[1] Bai R Liu Y Zhang L Dong W Bai Z Zhou M. Projections of future life expectancy in China up to 2035: a modelling study. Lancet Public Health. (2023) 8:e915–22. doi: 10.1016/S2468-2667(22)00338-3 37004714 PMC10188127

[2] Chen W Xu Y Zeng Y Xing G. A meta-analysis and systematic review based on perioperative management of elderly patients: is ciprofol an alternative to propofol? Eur J Clin Pharmacol. (2025) 81:111–21. doi: 10.1007/s00228-024-03782-7 39565391

[3] Yang H Deng HM Chen HY Tang SH Deng F Lu YG . The impact of age on propofol requirement for inducing loss of consciousness in elderly surgical patients. Front Pharmacol. (2022) 13:739552. doi: 10.3389/fphar.2022.739552 35418861 PMC8996377

[4] Yang H Fu Y Deng F Shao Y Lu YG Song JC. Median effective dose of dexmedetomidine inducing bradycardia in elderly patients determined by up-and-down sequential allocation method. Int J Med Sci. (2022) 19:1065–71. doi: 10.7150/ijms.71380 35813293 PMC9254370

[5] Akhtar SMM Fareed A Ali M Khan MS Ali A Mumtaz M . Efficacy and safety of Ciprofol compared with Propofol during general anesthesia induction: a systematic review and meta-analysis of randomized controlled trials (RCT). J Clin Anesth. (2024) 94:111425. doi: 10.1016/j.jclinane.2024.111425 38412619

[6] Hung KC Chen JY Wu SC Huang PY Wu JY Liu TH . A systematic review and meta-analysis comparing the efficacy and safety of ciprofol (HSK3486) versus propofol for anesthetic induction and non-ICU sedation. Front Pharmacol. (2023) 14:1225288. doi: 10.3389/fphar.2023.1225288 37818194 PMC10561285

[7] Ni T Zhou X Wu S Lv T Hu Y Gao Q . Hemodynamic impact of cipepofol vs propofol during anesthesia induction in patients with severe aortic stenosis: a randomized clinical trial. JAMA Surg. (2025) 160:763–70. doi: 10.1001/jamasurg.2025.1299 40397427 PMC12096327

[8] Ding YY Long YQ Yang HT Zhuang K Ji FH Peng K. Efficacy and safety of ciprofol for general anaesthesia induction in elderly patients undergoing major noncardiac surgery: a randomised controlled pilot trial. Eur J Anaesthesiol. (2022) 39:960–3. doi: 10.1097/EJA.0000000000001759 36214498

[9] Lu YF Wu JM Lan HY Xu QM Shi SQ Duan GC. Efficacy and safety of general anesthesia induction with ciprofol in hip fracture surgery of elderly patients: a randomized controlled trial. Drug Des Devel Ther. (2024) 18:3951–8. doi: 10.2147/DDDT.S475176 39247794 PMC11380857

[10] Teng Y Ou M Wang X Zhang W Liu X Liang Y . Efficacy and safety of ciprofol for the sedation/anesthesia in patients undergoing colonoscopy: phase IIa and IIb multi-center clinical trials. Eur J Pharm Sci. (2021) 164:105904. doi: 10.1016/j.ejps.2021.105904 34116176

[11] Li X Yang D Li Q Wang H Wang M Yan P . Safety, pharmacokinetics, and pharmacodynamics of a single bolus of the gamma-aminobutyric acid (GABA) receptor potentiator HSK3486 in healthy chinese elderly and non-elderly. Front Pharmacol. (2021) 12:735700. doi: 10.3389/fphar.2021.735700 34512361 PMC8430033

[12] Hu Y Li X Liu J Chen H Zheng W Zhang H . Safety, pharmacokinetics and pharmacodynamics of a novel gamma-aminobutyric acid (GABA) receptor potentiator, HSK3486, in Chinese patients with hepatic impairment. Ann Med. (2022) 54:2769–80. doi: 10.1080/07853890.2022.2129433 36217101 PMC9559057

[13] Tao J Liu S Zhao YY Qi L Yan P Wu N . Pharmacokinetics, pharmacodynamics, and safety of ciprofol emulsion in Chinese subjects with normal or impaired renal function. Front Pharmacol. (2023) 14:1260599. doi: 10.3389/fphar.2023.1260599 38074142 PMC10704090

[14] Petkar S Bele A Priya V Bawiskar D. Pharmacological insights and clinical applications of ciprofol: a narrative review. Cureus. (2024) 16:e68034. doi: 10.7759/cureus.68034 39347129 PMC11432775

[15] Duan G Lan H Shan W Wu Y Xu Q Dong X . Clinical effect of different doses of ciprofol for induction of general anesthesia in elderly patients: a randomized, controlled trial. Pharmacol Res Perspect. (2023) 11:e01066. doi: 10.1002/prp2.1066 36811327 PMC9944862

[16] Lu M Liu J Wu X Zhang Z. Ciprofol: a novel alternative to propofol in clinical intravenous anesthesia? Biomed Res Int. (2023) 2023:7443226. doi: 10.1155/2023/7443226 36714027 PMC9879693

[17] Xue Z Liu X Qian W Yang N Pan Y Zhou Y . The median effective dose of ciprofol combined with sufentanil for inhibiting responses to gastroscope insertion in obese patients: a prospective, single-center study. Drug Des Devel Ther. (2025) 19:3577–87. doi: 10.2147/DDDT.S494972 40330816 PMC12051986

[18] Deng L Zhang C Tan M Zeng W Luo G Li P. Effective dosage of ciprofol for the induction of general anesthesia across diverse age groups in adults: a single-center, prospective, non-randomized sequential trial. J Pain Res. (2025) 18:2983–92. doi: 10.2147/JPR.S496223 40534616 PMC12175962

[19] Guo X Qiao Y Yin S Luo F Yi L Chen J . Pharmacokinetics and pharmacodynamics of ciprofol after continuous infusion in elderly patients. BMC Anesthesiol. (2025) 25:41. doi: 10.1186/s12871-025-02907-4 39871139 PMC11771128

[20] Wang HY Lo MT Chen KH Mandell S Chang WK Lin C . Strong early phase parasympathetic inhibition followed by sympathetic withdrawal during propofol induction: temporal response assessed by wavelet-based spectral analysis and photoplethysmography. Front Physiol. (2021) 12:705153. doi: 10.3389/fphys.2021.705153 34588990 PMC8473792

[21] Sekiguchi R Kinoshita M Kawanishi R Kakuta N Sakai Y Tanaka K. Comparison of hemodynamics during induction of general anesthesia with remimazolam and target-controlled propofol in middle-aged and elderly patients: a single-center, randomized, controlled trial. BMC Anesthesiol. (2023) 23:14. doi: 10.1186/s12871-023-01974-9 36624371 PMC9830695

[22] Gelinas C Arbour C. Behavioral and physiologic indicators during a nociceptive procedure in conscious and unconscious mechanically ventilated adults: similar or different? J Crit Care. (2009) 24:628.e7–e17. doi: 10.1016/j.jcrc.2009.01.013 19327961

[23] Coleman RM Tousignant-Laflamme Y Gelinas C Choiniere M Atallah M Parenteau-Goudreault E . Changes in the bispectral index in response to experimental noxious stimuli in adults under general anesthesia. ISRN Pain. (2013) 2013:583920. doi: 10.1155/2013/583920 27335878 PMC4893395

[24] McDougall SJ Bailey TW Mendelowitz D Andresen MC. Propofol enhances both tonic and phasic inhibitory currents in second-order neurons of the solitary tract nucleus (NTS). Neuropharmacology. (2008) 54:552–63. doi: 10.1016/j.neuropharm.2007.11.001 18082229 PMC2351956

[25] Wang X. Propofol and isoflurane enhancement of tonic gamma-aminobutyric acid type a current in cardiac vagal neurons in the nucleus ambiguus. Anesth Analg. (2009) 108:142–8. doi: 10.1213/ane.0b013e31818d8b79 19095842

[26] Liu Y Yu X Zhu D Zeng J Lin Q Zang B . Safety and efficacy of ciprofol vs. propofol for sedation in intensive care unit patients with mechanical ventilation: a multi-center, open label, randomized, phase 2 trial. Chin Med J. (2022) 135:1043–51. doi: 10.1097/CM9.0000000000001912 34924506 PMC9276409

